# Prevalence and Patterns of Potentially Inappropriate Prescribing by Brazilian Dentists for Pediatric Patients: A Cross‐Sectional Study

**DOI:** 10.1155/bmri/4302237

**Published:** 2025-10-29

**Authors:** Widla Emanuella Pereira Barreto Garcez, Fatemah Abdullah, Jennifer Reis-Oliveira, Mary Angela Tavares, Mauro Henrique Nogueira Guimarães Abreu

**Affiliations:** ^1^ Department of Community and Preventive Dentistry, School of Dentistry, Universidade Federal de Minas Gerais, Belo Horizonte, Minas Gerais, Brazil, ufmg.br; ^2^ Department of Health Policy and Health Services Research, Boston University Goldman School of Dental Medicine, Boston, Massachusetts, USA

**Keywords:** antibacterial agents, dentistry, drug prescription, patient safety, pediatrics, pharmacoepidemiology, psychotropic drugs

## Abstract

Pediatric drug prescriptions raise significant safety concerns, particularly when potentially inappropriate medications are involved. This study is aimed at identifying and evaluating the frequency of antimicrobial and psychotropic medications considered potentially inappropriate when prescribed by dentists to children and adolescents in Brazil. A cross‐sectional study was conducted using secondary data from the National System for Controlled Products Management (SNGPC) between December 2020 and November 2021. To assess the frequency of adverse drug reactions (ADRs), medications were classified according to the Key Potentially Inappropriate Drugs in Pediatrics (KIDs′ List). Data were organized in Excel and analyzed descriptively. Negative binomial regression models were used to estimate prevalence ratios (PRs) with 95% CI, adjusted for age and gender. Analyses were performed in SPSS Version 29.0. A total of 204,026 dental prescriptions for individuals under 18 years of age were recorded over the 12 months. The overall prescription rate of risky medication was 2.2 per 10,000 pediatric patients. Among the risky prescriptions, antibiotics were the most frequently dispensed, accounting for 41.2%, with azithromycin being the most commonly prescribed (68.5%). The Southeast region showed the highest frequency of both total and risky prescriptions. Males and neonates/infants received a higher number of risky prescriptions. These findings underscore the urgent need for personalized clinical guidelines for pediatric dental prescribing in Brazil. Promoting the rational use of medications in this population is essential to minimizing ADRs and improving patient safety.

**Summary**

• Brazilian dentists could prescribe antimicrobials and psychotropic drugs for children and adolescents.
• Analysis from all dental prescriptions from secondary dataset (National System for Controlled Products Management) in Brazil was carried out from December 2020 to November 2021.
• From 204,026 dental prescriptions, 6939 (3.4%) involved a risky medication for individuals under 18 years old.
• These findings underscore the urgent need for personalized clinical guidelines to pediatric dental prescribing in Brazil.
• Children and teenagers sometimes receive prescriptions from dentists, but not all medications are safe for their age.
• We looked at all controlled medications prescribed by dentists to patients under 18 years old across Brazil for 1 year. Our goal was to find out how often dentists prescribed drugs that may not be recommended for this population.
• We used the KIDs′ List to check which medications could be risky. We found 204,026 prescriptions, and approximately 7000 of them included drugs that may not be appropriate for children. Most of these were antibiotics, especially azithromycin.
• Boys and newborns/babies received more risky prescriptions than others, and the Southeast region had the highest number overall. These results show that dentists may need more support and guidance when prescribing medicine to young patients. Using the right medication helps to keep children safe and prevents side effects.

## 1. Introduction

The safety of pharmacological interventions in the pediatric population constitutes a critical issue within child healthcare and has emerged as a growing priority in public health research. Despite significant progress in pharmacotherapy, children remain especially susceptible to adverse drug reactions (ADRs) due to developmental differences in drug metabolism, complexities in dosing, and the frequent use of off‐label medications [1]. In response to these challenges, the Pediatric Pharmacy Association (PPA) developed the Key Potentially Inappropriate Drugs in Pediatrics (KIDs′ List), which delineates 67 active pharmaceutical ingredients that should be either avoided or prescribed with caution in specific pediatric subpopulations [2]. This evidence‐based instrument provides a foundation for identifying potentially harmful pharmacological agents and fostering safer prescribing practices in pediatric care. In Brazil, the National Health Surveillance Agency (ANVISA) monitors the inflow and outflow of antimicrobials and psychotropic drugs dispensed by private pharmacies and drugstores across the country [3, 4].

Significant disparities in socioeconomic conditions, healthcare infrastructure, and medication access characterize Brazil′s five geographic regions (North, Northeast, Southeast, South, and Midwest). These variations have a profound impact on prescribing patterns and health outcomes within pediatric populations. Research indicates that regions with poorer socioeconomic standing, such as the North and Northeast, encounter significant impediments to rational drug use, encompassing restricted access to pediatric specialists, diminished availability of essential medicines, and deficient pharmaceutical services [5]. In contrast, the Southeast and South regions possess superior healthcare resources and pharmacovigilance programs, although they concurrently report heightened rates of polypharmacy and off‐label prescriptions [6]. These interregional discrepancies reinforce the importance of integrating a contextualized framework when evaluating pediatric prescribing practices throughout Brazil.

The need for such guidance is especially urgent given the widespread—and often inappropriate—use of medications in pediatric care. Dentists play a significant role in this context, accounting for an estimated 10% of all global antibiotic prescriptions [7]. However, a considerable proportion of these prescriptions are either unnecessary or inconsistent with current clinical guidelines [8]. A cross‐sectional study in Brazil found that only 13.2% of dental antibiotic prescriptions for prophylaxis adhered to established protocols regarding drug selection, dosage, and duration [9]. Antibiotics should be prescribed for dental infections only when systemic involvement is evident, and even then, treatment should be limited to monotherapy with amoxicillin or phenoxymethylpenicillin in nonallergic patients [10]. Inappropriate antibiotic use not only exposes children to preventable ADRs but also contributes to the global threat of antimicrobial resistance (AMR). Without significant changes in prescription and consumption patterns, AMR could result in up to 10 million deaths annually by 2050 and a cumulative economic loss of at least USD 60 trillion [11].

Beyond antibiotics, some psychotropic drugs could be prescribed by dentists for outpatient care. Children often experience fear and anxiety during dental visits, and nonpharmacologic strategies can alleviate anxiety [12]. For the management of dental pain, the American Academy of Pediatric Dentistry (AAPD) recommends that opioid use in pediatric dental patients should be rare, as it increases the risk of persistent use, misuse, and diversion, especially following procedures such as dental extractions [13]. Nevertheless, in the United States in 2019, dentists accounted for 38% of all opioid prescriptions filled by individuals aged 0–21 years [14]. Moreover, dentists may inappropriately refill prescriptions issued by other providers for nondental indications, such as amphetamines, opioids, benzodiazepines, sedatives, and off‐label antipsychotics or stimulants [15].

The discussion of potentially inappropriate prescriptions (PIPs) must expand beyond antimicrobials and psychotropics to encompass the broader range of drug classes listed in the KIDs′ List, including antihistamines, gastrointestinal agents, cardiovascular drugs, and analgesics [2, 16]. Recent studies highlight that the inappropriate use of medications such as first‐generation antihistamines, codeine, and certain proton pump inhibitors can lead to significant and preventable harm in children, including respiratory depression, sedation, and metabolic disturbances [17, 18]. Therefore, a more comprehensive evaluation of PIPs across all drug classes is essential to capture the scope of risks in pediatric care and to inform targeted interventions for safer prescribing practices in dentistry and beyond.

Given that dentists in Brazil contribute to these prescribing trends, this study is aimed at identifying and evaluating the frequency of potentially inappropriate antimicrobial and psychotropic medications prescribed by dentists to children and adolescents in Brazil. Using the KIDs′ List as a reference to assess the risk of ADRs, we leveraged national surveillance data from the National System for Controlled Products Management (SNGPC) to describe and analyze prescribing patterns by age, sex, and geographical region and to highlight the public health implications of inappropriate prescribing in pediatric dental care.

## 2. Methods

A cross‐sectional study from secondary data analysis from the SNGPC in Brazil, from December 2020 to November 2021, was conducted, the latest 12‐month period update of the database. The SNGPC is an electronic platform managed by the ANVISA that monitors the inflow and outflow of psychotropic drugs and antimicrobials dispensed by private pharmacies and drugstores across the country [3, 4]. The database includes information such as year, month, state, and municipality of sale, medication name, drug description and formulation, quantity sold, professional′s licensing board and state of registration, prescription type, International Classification of Disease (ICD) code, patient sex, age, and age unit (both in year and month).

In the first stage of the analysis, all individuals aged 0–17 years who received prescriptions by dentists were selected from the SNGPC database across all 26 states and the Brazilian Federal District. Prescriptions with missing data on the medication name were excluded from the sample. To identify and evaluate the risk of medications associated with high risk for ADRs, we used the “KIDs′ List.” The KIDs′ List was thought of and created by a group of pediatric pharmacists designed from the PPA to evaluate the medical literature and compare a list of drugs that should be “avoided” or “used with caution” in all or part of the pediatric population. The method used for KID construction is robust and involves an extensive literature review and a panel discussion, facilitating the compilation of the first iteration of a list of drugs [2]. In the KIDs′ List, it is possible to identify the age group for which a given medication should be used with caution or avoided. In our study, this information was used to determine the risk associated with each prescription (0 = nonrisky; 1 = risky). The primary outcome of the study was to assess the frequency of KIDs′ dental prescription of psychotropic drugs and antimicrobials. The name of each drug at SNGPC was confirmed using Anatomical Therapeutic Classification (ATC) from World Health Organization (WHO) Collaborating Centre for Drug Statistics Methodology [19].

The data was organized using Microsoft Excel for Windows. Initially, a descriptive analysis was conducted by calculating the rate of prescribed drugs among the Brazilian population under 18 years old and the proportion of KIDs′ drugs among the total drugs prescribed by dentists during this period. The Brazilian population was estimated by the Brazilian Institute of Geography and Statistics (IBGE, 2024) [20]. Subsequently, a stratified analysis of the frequency of KID‐listed drugs was performed according to age classification, gender, and geographical region. For the covariate age, considering that KIDs′ List does not provide a highly precise age classification, we adopted a custom age classification, guided by the recommendations of the American Academy of Pediatrics (AAP) and the criteria proposed by Williams et al. [21, 22]. Neonates were those from birth to 28 days. Individuals up to 12 months were classified as infants. Children from 1 to 5 years old were in early childhood. From 5 to 11 years old were middle childhood. Early adolescents were the group from 11 to 14 years and middle adolescents from 14 to 17. For statistical analysis, the six original age groups were collapsed into three, since one of them presented no risk prescriptions, which would compromise the validity of the analysis. Thus, the age groups considered were neonates/infants, childhood (early and middle childhood), and adolescents (early and middle adolescents). Gender was dichotomized (male and female), and the geographical region was one of the Brazilian five regions (South, Southeast, Central‐West, Northeast, and North).

Descriptive statistics were conducted through the calculation of proportions. The dependent variable analyzed was prescription risk, categorized as risky or nonrisky. Negative binomial regression models were carried out to estimate both unadjusted and adjusted prevalence ratios (PRs) with their respective 95% confidence intervals (CIs) and *p* values, considering the individual factors′ age and gender as explanatory variables. We categorized pediatric patients into three age groups to analyze prescription trends across developmental stages. To evaluate the goodness of fit of the final model, we used the ratio between residual deviance and degrees of freedom, as well as the chi‐squared test of the residual deviance [23, 24]. All statistical analyses were carried out in SPSS Version 29.0.

## 3. Results

Among the 67 active ingredients listed in the KIDs′ List, 17 were identified in the final sample. The total number of dental prescriptions in this 12‐month study was 204,026. After excluding those with missing data (*n* = 166), 6939 (3.4%) involved a risky medication from the KIDs′ List. The prescription rate of these risky medications for individuals under 18 years old in Brazil was 2.2 prescriptions by 10,000 pediatric patients. The Top 10 most prescribed active ingredients from the risky prescriptions were azithromycin (68.5%), followed by codeine (22.6%), sulfamethoxazole (2.7%), tetracycline (1.5%), nitrofurantoin and chloramphenicol (each accounting for 1.2%), ceftriaxone (0.9%), betamethasone and silver sulfadiazine (each accounting for 0.7%), and gentamicin (0.3%) (Table 1).

**Table 1:** Frequency of medication from KIDs′ List among Brazilian individuals from 0 to 17 years old, 2020–2021.

**Active ingredient**	**Frequency** ^**a**^
1. Azithromycin	4752
2. Codeine	1570
3. Sulfamethoxazole	190
4. Tetracycline	102
5. Nitrofurantoin	87
6. Chloramphenicol	81
7. Ceftriaxone	60
8. Betamethasone	46
9. Silver sulfadiazine	46
10. Gentamicin	20
11. Lamotrigine	10
12. Midazolam	7
13. Olanzapine	4
14. Ivermectin	3
15. Chlorpromazine	1
16. Haloperidol	1
17. Trifluoperazine	1

^a^Some prescriptions contain more than one active ingredient; therefore, the total presented in this table is 6981.

Compared with adolescents, neonates/infants showed a markedly higher prevalence of risky prescriptions (adjusted PR: 26.01; 95% CI: 23.49–28.80; *p* < 0.001). In childhood, the prevalence of risky prescriptions was 1.80 times higher (95% CI: 1.54–2.10; *p* < 0.001) than that of adolescents. Males accounted for the most risky prescriptions (62.5%), whereas females represented 37.5% of cases. After adjustment, males had a 12% higher prevalence of risky prescriptions compared with females (adjusted PR: 1.12; 95% CI: 1.10–1.14; *p* < 0.001) (Table 2). The chi‐squared test of the residual deviance indicated that the model fit well (*p* = 1.0). The final value obtained was 1, indicating an adequate fit of the model. The Southeast Region showed the highest prevalence of KIDs′ dental prescriptions (51.5%), as well as the highest proportion of risky prescriptions (55.0%), followed by the South, Central‐West, Northeast, and North regions (Table 3).

**Table 2:** Distribution of total and KIDs′ prescriptions and results of unadjusted and adjusted negative binomial regression among dentists, by age and gender.

**Age group (total of prescriptions; %)**	**Number of risky prescriptions (%)**	**Nonadjusted PR (CI 95%)**	**p** **value**	**Adjusted PR (CI 95%)**	**p** **value**
Neonates/infants (*N* = 37,156; 18.3%)	6323 (91.1%)	26.39 (23.83–29.22)	< 0.001	26.01 (23.49–28.80)	< 0.001
Childhood (*N* = 45,569; 22.3%)	264 (3.8%)	1.82 (1.56–2.12)	< 0.001	1.80 (1.54–2.10)	< 0.001
Adolescents (*N* = 121,301; 59.4%)	352 (5.1%)	1		1	
Gender					
Male (*N* = 103,932; 51.0%)	4335 (62.5%)	1.44 (1.39–1.49)	< 0.001	1.12 (1.10–1.14)	< 0.001
Female (*N* = 100,094; 49.0%)	2604 (37.5%)	1		1	
Total (*N* = 204,026; 100.0%)	6939 (100.0%)				

Abbreviation: PR, prevalence ratio.

**Table 3:** Distribution of total and KIDs′ prescriptions by Brazilian geographical regions.

**Geographical region**	**Total dental prescriptions**	**Risky dental prescriptions**
South	42,210 (20.7%)	1668 (24.0%)
Southeast	105,143 (51.5%)	3813 (55.0%)
Central‐West	17,905 (8.8%)	653 (9.4%)
Northeast	27,115 (13.3%)	460 (6.6%)
North	11,653 (5.7%)	345 (5.0%)
Total	204,026 (100.0%)	6939 (100.0%)

## 4. Discussion

Notably, Brazilian children received dental prescriptions of risky drugs, involving medications with potential safety concerns. Beyond quantifying this, interpreting their distribution provides insights into pediatric prescribing practices. Understanding the distribution of these prescriptions and the reason why the medications were prescribed is crucial for informing regulatory measures and promoting safer medication practices for pediatric patients.

When analyzing prescriptions classified as high risk, a significant gender disparity was observed. Male patients accounted for most of these prescriptions and showed a higher prevalence compared with females. This finding indicates that gender is an important determinant of exposure to PIPs in dental practice. These findings may be attributed to the observation that the most frequently utilized therapeutic drug classes in pediatrics, such as antibiotics, are more commonly prescribed to males than to females. This pattern is evident in the study by Ferrajolo et al. [25], which reported a higher rate of antibiotic prescriptions among male individuals. A plausible reason for these gender‐related patterns in drug utilization may be the indications for which these medications are typically prescribed [25]. Nevertheless, our results contrast with those reported by Lee and Kim [26], who observed a higher prevalence of antibiotic prescriptions among females compared to male children and adolescents in South Korea.

The higher prevalence of risky prescriptions in neonates/infants can be explained by a combination of factors. Newborns and babies exhibit immaturity in the hepatic and renal systems, which compromises drug metabolism and excretion, thus increasing susceptibility to adverse reactions and drug interactions [17]. Furthermore, many medications used in clinical practice for this age group do not have formal approval, being used off‐label, which contributes to the increased risk of PIPs [27]. Regarding the clinical management of neonates/infants, it is largely complex, which elevates the probability of a prescription being considered a risky prescription [28]. These aspects highlight the need for specific protocols and further studies focusing on drug safety in neonates/infants, aiming to reduce adverse events and promote evidence‐based practices [29]. Our results indicate a higher frequency of both total and risky dental prescriptions in the Southeast Region, which, according to the 2022 IBGE Census [30], concentrates most of Brazil′s population (41.8%) and individuals aged 0–17 years (38.1%). Similarly, the South, the third most populous region, concentrates 14.7% of the total population and 13.7% of the 0–17 age group. As more developed and densely populated regions, the Southeast and South also offer expanded access to dental services [31], especially in the private sector. Furthermore, these regions have a larger number of dental professionals and undergraduate dentistry courses [32], which contributes to the high volume of prescriptions. This context may help explain the higher risky prescriptions identified in these regions.

During the study period in Brazil, antibiotics accounted for the largest proportion of risky prescriptions issued by dentists to children and adolescents. Other drug classes including analgesics, corticosteroids, topical antimicrobials, anticonvulsants, benzodiazepines, and antiparasitic agents were dispensed at comparable rates. Antibiotics are commonly used in dentistry for both therapeutic and prophylactic purposes. Azithromycin, for example, is prescribed to treat bacterial infections, particularly in patients with allergies to penicillin or ampicillin. In pediatric cases, it is considered an appropriate choice due to its long half‐life, which reduces the frequency of administration and facilitates treatment adherence [33].

In the present study, azithromycin was the most frequently prescribed risky active ingredient (68.5%). This finding must be highlighted, as azithromycin is more expensive than amoxicillin, which may impose a higher financial cost for the patient or the public healthcare system. Moreover, its prolonged use can lead to the growth of azithromycin‐resistant bacteria, and in neonates, it has been associated with hypertrophic pyloric stenosis [2, 34, 35]. Codeine was the second most frequently used active ingredient in risky prescriptions, representing 22.6% of prescriptions and is associated with serious adverse effects, including respiratory depression and, in severe cases, death; its use in children is strongly discouraged. Sulfamethoxazole (2.7%) should also be avoided, particularly in neonates; it may lead to kernicterus, a severe neurological condition caused by the accumulation of bilirubin in the brain [2]. These drugs can result in serious complications in pediatric populations and must be prescribed with caution. Dentists should be more attentive and responsible in prescribing practices, regardless of the patient′s age or the medication involved.

These findings emphasize the necessity of strengthening monitoring systems and refining prescribing protocols, particularly for neonates and infants, where the use of high‐risk medications is most prevalent. Neonates represent one of the most vulnerable pediatric populations to ADRs. In the study by Kaguelidou et al. [35], which assessed the occurrence of ADRs in neonatal patients, 56% of the 1688 neonates who experienced an ADR presented with serious reactions.

Our findings suggest a lack of knowledge of professionals regarding the prescription of medications for children and adolescents, potentially leading to serious complications in this age group. This problem, specifically in relation to antibiotic prescribing practices, has also been identified in studies conducted in other countries [36, 37], highlighting the need for global and national public policies and training to minimize unnecessary risks to the population. This lack of knowledge can result in inappropriate or even unnecessary prescriptions. AMR has emerged as a pressing public health problem, and both overprescribing and the improper use of antibiotics significantly contribute to this. Several reports by the WHO have identified the improper and excessive use of antibiotics as the leading risk factor in the global escalation of AMR [38].

This study has some limitations, such as it was based on a secondary database whose most recent update was in November 2021, meaning that the prescription frequency of these medications may have changed since then. We also encountered a considerable amount of missing data, particularly regarding the ICD, which, if available, could have provided insight into the indications for these prescriptions. However, to the best of our knowledge, this is the first representative study to evaluate antimicrobial and psychotropic prescriptions among Brazilian children and adolescents.

## 5. Conclusion

Antibiotics were the most frequently dispensed medications prescribed by dentists to children and adolescents in Brazil during the 12‐month follow‐up period. Risky prescriptions were more common among males and neonates/infants. Furthermore, the Southeast and South regions accounted for the highest rates of risky prescriptions in this population. This study reinforces the need for public policies and evidence‐based guidelines to reduce inappropriate prescribing and ensure the safety of pediatric patients.

## Conflicts of Interest

The authors declare no conflicts of interest.

## Funding

This work was supported by CAPES (Foundation for the Improvement of Higher Education Personnel, 001), CNPq (National Council for Scientific and Technological Development, 305806/2023‐8), FAPEMIG (Minas Gerais State Research Foundation, APQ‐00711‐23), and Brazilian Health Surveillance Agency (ANVISA) for the development of the SNGPC system.

## Data Availability

The data that support the findings of this article are available from the corresponding author on reasonable request.
